# Neutrophil-to-Lymphocyte Ratio for Predicting Loss of Response to Infliximab in Ulcerative Colitis

**DOI:** 10.1371/journal.pone.0169845

**Published:** 2017-01-11

**Authors:** Yu Nishida, Shuhei Hosomi, Hirokazu Yamagami, Tomomi Yukawa, Koji Otani, Yasuaki Nagami, Fumio Tanaka, Koichi Taira, Noriko Kamata, Tetsuya Tanigawa, Masatsugu Shiba, Kenji Watanabe, Toshio Watanabe, Kazunari Tominaga, Yasuhiro Fujiwara

**Affiliations:** 1 Department of Gastroenterology, Osaka City University Graduate School of Medicine, Osaka, Japan; 2 Department of Gastroenterology, Osaka City General Hospital, Osaka, Japan; Kurume University School of Medicine, JAPAN

## Abstract

**Objectives:**

Neutrophil-to-lymphocyte ratio (NLR) has been used to determine the outcome in malignancies and coronary heart disease. Some reports considered the value of NLR as a predictor of response to infliximab in patients with Crohn’s disease or rheumatoid arthritis; however, no similar studies have been reported for ulcerative colitis (UC). This study aimed to evaluate the clinical significance of the baseline NLR in patients with UC treated by infliximab.

**Materials and Methods:**

Patients with moderate-to-severe active UC who received the first infliximab infusion in our hospital between 2010 and 2015, who showed clinical response during the induction period, were retrospectively evaluated for long-term outcomes and risk factors for loss of response (LOR) during infliximab maintenance therapy. Baseline inflammatory markers including NLR were measured within one week before the initiation of infliximab.

**Results:**

Fifty-nine patients with moderate-to-severe active UC started treatment with infliximab and 37 patients (62.7%) experienced clinical response after induction therapy. Fourteen of 37 patients on maintenance therapy lost the response during follow-up. Baseline NLR of patients with LOR was significantly higher than in patients with sustained response. The NLR cut-off value of 4.488 was predictive of LOR, using receiver operating characteristic analysis (sensitivity: 78.6%, specificity: 78.3%). A univariate analysis revealed a significant relationship between relapse-free survival and the NLR (*P* = 0.018). Multivariate analysis indicated the NLR as an independent prognostic factor for LOR (hazard ratio = 3.86, 95% confidence interval: 1.20–12.4, *P* = 0.023).

**Conclusions:**

Baseline NLR is a useful prognostic marker in patients with moderate-to-severe active UC treated with infliximab, and may contribute to appropriate use of infliximab.

## Introduction

Ulcerative colitis (UC) is a chronic inflammatory disease of the colon with unknown etiology, and is characterized by a typical natural course with recurrent flares of mucosal inflammation. Most patients with UC can be successfully treated with 5-aminosalicylates, corticosteroids and immunomodulators [1]. However, those patients requiring corticosteroids at any point face a severe disease course over time, with a high requirement of immunosuppressant treatment and colectomy, resulting in a high cost for the health care system [2, 3]. Treatment with the anti-tumor necrotic factor (TNF) antibody infliximab has proven effective for the induction and maintenance of a remission in moderate to severe UC [4]. However, the loss of response (LOR) is a common clinical problem, with the incidence ranging between 23%–46% at 12 months after anti-TNF initiation [5]. Therefore, it is necessary to identify biomarkers for predicting the LOR to infliximab in UC.

Markers based on systemic inflammation, such as C-reactive protein (CRP), TNFα, interleukin-6, or white blood cell (WBC) count have been reported to be useful in predicting the prognosis in patients with inflammatory disease including inflammatory bowel disease [6]. Neutrophil-to-lymphocyte ratio (NLR) has also been reported as a useful predicting factor for various types of cancer [7–14], rheumatoid arthritis [15], and coronary heart disease [16–19]. Furthermore, several studies have reported the association between NLR and the activity of UC, with optimum NLR cut-off values of 2.16 to 2.47. [20–23]. With regard to infliximab, one study reported that a pre-treatment NLR lower than 4.068 predicts a sustained response to a 52-week course of infliximab therapy in Crohn’s disease [24], whereas another study reported that baseline NLR did not predict the response to biological agents in rheumatoid arthritis [25]. However, to the best of our knowledge, no study has analyzed the predictive value of the NLR as a prognostic marker in patients with UC receiving infliximab. Therefore, the objective of our study was to evaluate the clinical significance of the pre-treatment NLR as a prognostic factor in patients with UC receiving infliximab. We evaluated the long-term outcome of infliximab and analyzed the prognostic factors for the outcome, including the baseline inflammatory markers.

## Materials and Methods

### Patients

All patients with moderate-to-severe active UC who received the first infusion of infliximab in our hospital between June 2010 and November 2015, and achieved clinical response during induction period were enrolled in the study. Infliximab was administered per the recommended dosage regimen: induction with 5 mg/kg at week 0, 2 and 6, and a maintenance dosage every 8 weeks. The diagnosis of UC was based on clinical, endoscopic and histopathological findings. Demographic, clinical, and laboratory data were obtained from the medical records. The pre-treatment markers of systemic inflammation were measured within one week before the initiation of infliximab.

### Evaluation

Response evaluations were performed at week-2, week-6, and thereafter every 8 weeks. All patients were followed up with a physical examination and a blood test. The differential WBC count was analyzed using an XE-5000 hematology analyzer (Sysmex, Kobe, Japan), as per the manufacture’s protocol. In each case, the NLR was calculated from a blood sample by dividing the absolute neutrophil count with the absolute lymphocyte count. Patients were followed from the first infusion of infliximab to relapse, or until the end of May 2016. Patients with infliximab withdrawal during deep remission in the maintenance period (per the physician’s decision) were followed up until the cessation of infliximab.

### Study endpoint

The primary outcome measure of this study was LOR to infliximab.

### Definitions

The partial Mayo score [26] was used to assess disease activity. Moderate-to-severe active disease was defined as a partial Mayo score ≥5. Severe UC was defined as a partial Mayo score ≥7. An LOR to infliximab was characterized by a clinical relapse requiring infliximab dose optimization or secondary alternative therapies among responders during the maintenance periods [27–29], whereas a sustained response to infliximab was defined as absence of LOR over the follow-up period among responders. Responders were defined as patients who achieved a clinical response at the end of the induction period (at week 14), without secondary alternative therapies such as tacrolimus, corticosteroids, or proctocolectomy. Clinical response was defined as a partial Mayo score reduction of ≥3 points, accompanied by a decrease of at least 30% from the baseline, and a decrease in the rectal bleeding subscore of ≥1, or an absolute rectal bleeding subscore of 0 or 1 [30].

### Statistical analysis

Continuous variables were summarized with the median and the interquartile range (IQR). Receiver operating characteristics (ROC) curves were plotted to calculate the area under the ROC curve. The differences in the clinical characteristics were compared using either the chi-square test or Fisher’s exact test for categorical variables, and the Mann-Whitney U test for continuous variables. Differences in continuous variables between baseline dataset and dataset at 14 week were compared by Wilcoxon rank sum test. Correlations were calculated using the Spearman’s rank correlation. The prognostic factors for LOR were evaluated for cumulative relapse-free rate among the responders. The cumulative relapse-free rate was illustrated with a Kaplan-Meier plot. Differences in the survival curves were assessed with the log–rank test. A multivariate analysis was performed according to the Cox regression model. The data were presented as hazard ratios (HR) with 95% confidence intervals (CI). A *P*-value less than 0.05 was regarded as statistically significant. All statistical analyses were performed with EZR (Saitama Medical Center, Jichi Medical University), a graphical user interface for R (The R Foundation for Statistical Computing, version 2.13.0). More precisely, it is a modified version of R commander (version 1.6–3) that includes statistical functions frequently used in biostatistics.

### Ethical considerations

This study was approved by the ethics committee of Osaka City University Hospital (No. 3488). The ethics committee granted exemption for written informed consent to this study because the analysis used anonymized clinical data that were retrospectively obtained after each patient agreed to treatment by written consent. Nevertheless, all patients were notified of the content and information in this study and were given the opportunity to refuse participation. None of the patients refused participation. This procedure followed the Ethical Guidelines for Medical and Health Research Involving Human Subjects established by the Ministry of Education, Culture, Sports, Science and Technology and the Ministry of Health, Labour and Welfare in Japan.

## Results

### Study subjects

**Fig 1:**
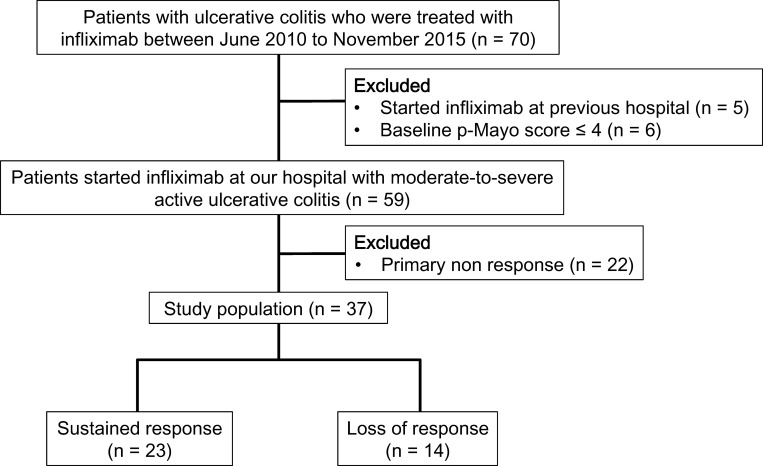
Flow chart of the study population.

Fifty-nine patients with UC started treatment with infliximab, and 37 patients (62.7%) obtained a clinical response after the induction therapy (Fig 1). We retrospectively included these 37 patients (14 males, 23 females) in the subsequent analysis. The demographic particulars of the patients are summarized in Table 1. Males accounted for 37.8% of the patient population. The median age was 44.2 years with IQR from 35.8 to 53.4 years. The median duration from UC diagnosis to admission was 5.2 years with IQR from 2.5 to 10.0 years. At the time of infliximab induction, concomitant therapies with mesalamine, corticosteroids, immunomodulators (azathioprine or 6-mercaptopurine), tacrolimus, and cytapheresis were used in 91.9%, 37.8%, 45.9%, 10.8%, and 27.0% of patients, respectively. The median disease activity value at the baseline was 7, with IQR from 5 to 7, using the partial Mayo score. During a median follow-up period of 19.1 months with IQR from 8.4 to 32.0 months, 14 of 37 patients on maintenance therapy lost response during follow-up period (Fig 1). No patient had malignancies, two had a history of aortic valve replacement and took warfarin, and one had a history of renal artery angioplasty; however, no patients had symptoms related to cardiovascular disease.

**Table 1:** Baseline characteristics of the study population.

	all patients
Number of patients	37
Gender: male/female	14/23
Age at diagnosis, median (interquartile range)	35.3 (26.7–43.9) years
Age at start of infliximab, median (interquartile range)	44.2 (35.8–53.4) years
Disease duration, median (interquartile range)	5.2 (2.5–10.0) years
UC location: Left-sided colitis/Pancolitis	10/27
Response to corticosteroids	
Dependent, n (%)	18 (48.6%)
Resistant, n (%)	14 (37.8%)
Concomitant therapies, n (%)	
Mesalamine	34 (91.9%)
Corticosteroids	14 (37.8%)
Immunomodulators (azathioprine or 6-mercaptopurine)	17 (45.9%)
Tacrolimus	4 (10.8%)
Cytapheresis	10 (27.0%)
partial Mayo score, median (interquartile range)	7 (5–7)
WBC (/μL), median (interquartile range)	6100 (5000–7800)
Hemoglobin (g/dL), median (interquartile range)	11.5 (10.8–12.6)
Platelet (×10^4^/μL), median (interquartile range)	28.5 (21.2–31.9)
Albumin (g/dL), median (interquartile range)	3.80 (3.50–4.10)
CRP (mg/dL), median (interquartile range)	0.27 (0.04–0.77)
NLR, median (interquartile range)	3.19 (1.82–5.52)

UC: ulcerative colitis; WBC: white blood cell; CRP: C-reactive protein; NLR: neutrophil-to-lymphocyte ratio.

### Predictive factors for LOR in responders

**Fig 2:**
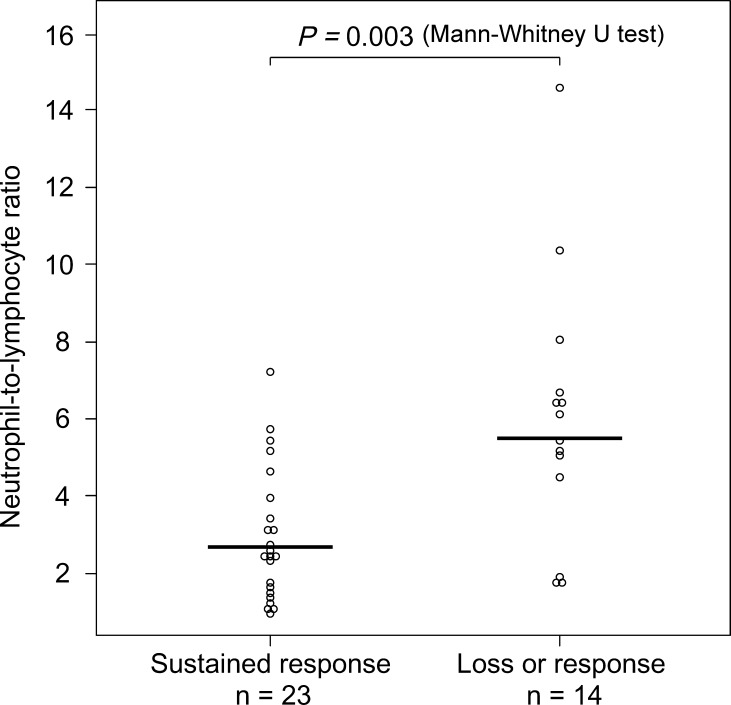
Comparison between pre-treatment neutrophil-to-lymphocyte ratios (NLR) of patients with sustained response and patients with loss of response. The horizontal bar represents median value.

**Fig 3:**
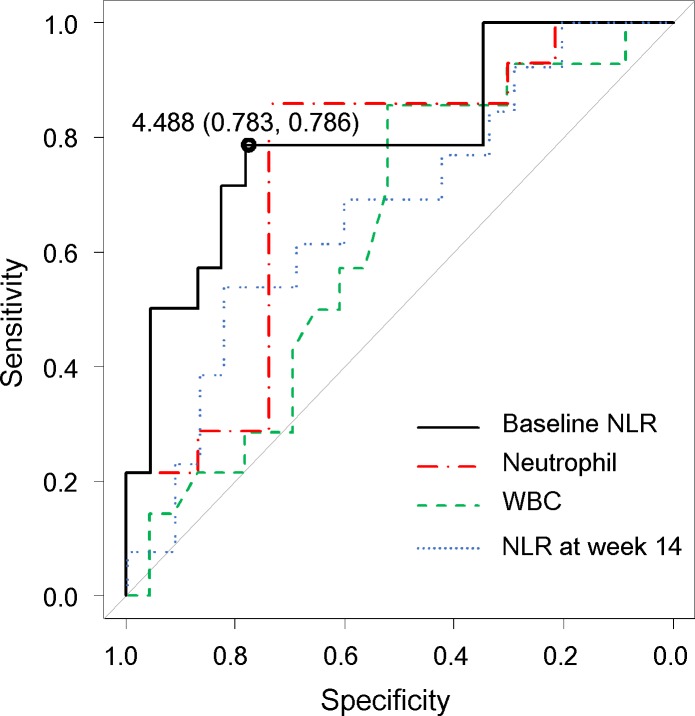
Receiver operating characteristic curve comparing the baseline neutrophil-to-lymphocyte ratio (NLR), NLR at week 14, white blood cell (WBC) count, and the neutrophil count for predicting LOR to infliximab in patients with ulcerative colitis (UC). Baseline NLR: area under the curve (AUC) = 0.798, NLR at week 14: AUC = 0.702, WBC count: AUC = 0.627, neutrophil count: AUC = 0.736.

**Fig 4:**
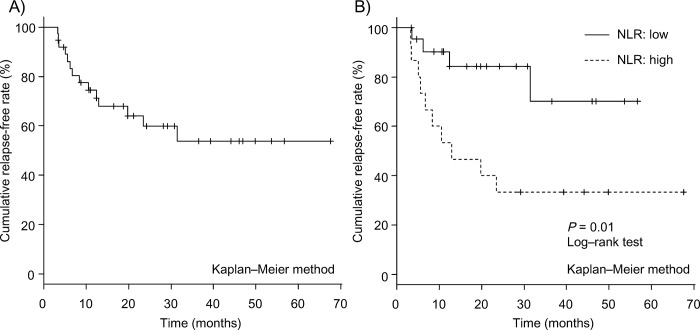
Relapse-free survival. Overall relapse-free survival in 37 responders with infliximab (A), relapse-free survival based on neutrophil-to-lymphocyte ratio (NLR) at the start of infliximab, the overall survival rate was significantly worse in the high NLR group than in the low NLR group (*P* = 0.01) (B).

Table 2 shows comparison of patients’ demographic variables at the baseline between the two groups (the sustained response group and the LOR group). The NLR in patients with LOR (median: 5.78, 95% CI: 4.62–6.58) was significantly higher than in patients with sustained response (median: 2.46, 95% CI: 1.57–3.68) (*P* = 0.003) (Fig 2), though no significant differences were noted in baseline NLR between responders and non-responders (*P* = 0.707). After administration of infliximab, no significant differences were found between baseline NLR and NLR at the start of the maintenance period (at week 14) among patients with loss of response, patients with sustained response, and non-responders (patients with loss of response: *P* = 0.173, patients with sustained response: *P* = 0.427, non-responders: *P* = 0.063, Wilcoxon rank sum test) (S1 Fig). Although the WBC and neutrophil counts and the NLR at the start of the maintenance period (at week 14) were also higher in patients with LOR than in patients with sustained response, the baseline NLR had the highest predictive value, with an area under the ROC curve of 0.798; in contrast, WBC and neutrophil counts and the NLR at week 14 had lower predictive value, with areas under the ROC curve of 0.627, 0.736, and 0.702, respectively (Fig 3). The ROC curve analysis showed that the best cut-off values for the NLR were greater than 4.488 (sensitivity: 78.6%, specificity: 78.3%). We therefore set 4.488 as the cut-off value, and the patients were classified into high-NLR (n = 15) and low-NLR (n = 22) groups. The relapse-free survival rate was significantly worse in the high NLR group than in the low NLR group (*P* = 0.01) (Fig 4). The correlations between the relapse-free survival and the various clinical factors are shown in Table 3. According to the results of a univariate analysis, relapse-free survival exhibited a significant relationship with the NLR (*P* = 0.018). As concomitant therapies may affect NLR, we evaluated this association in patients with and without concomitant drug treatment (S1 Table). As expected, patients receiving corticosteroid treatment showed a tendency toward a higher baseline NLR than those not receiving corticosteroid treatment in this study, although no correlation was noted between corticosteroid dosage and the baseline NLR (S2 Fig). On the other hand, no significant correlations were noted between baseline NLR and baseline partial Mayo score (S3 Fig). Therefore, to elucidate the influence of corticosteroid treatment, the variables identified by a univariate analysis with *P*-value less than 0.2, and a concomitant corticosteroid treatment at the start of infliximab were included in the multivariate analysis. The multivariate analysis identified the NLR (HR = 3.86, 95% CI: 1.20–12.4, *P* = 0.023) as an independent prognostic factor for LOR.

**Table 2:** Variables associated with loss of response to infliximab in patients with ulcerative colitis.

	Sustained response	Loss of response	*P*-value
Number of patients	23	14	
Gender: male/female	8/15	6/8	0.732
Age at diagnosis, median (interquartile range) *yr*.	36.1 (27.9–43.7).	35.1 (26.8–46.1)	0.826
Age at start of infliximab, median (interquartile range) *yr*.	44.5 (34.2–53.9)	42.4 (35.8–50.9)	0.778
Disease duration, median (interquartile range) *yr*.	5.2 (2.6–8.9)	5.3 (2.6–10.1)	0.963
UC location: Left-sided colitis/Pancolitis	6/17	4/10	1
Response to corticosteroids			
Dependent, n (%)	11 (47.8%)	7 (50.0%)	1
Resistant, n (%)	8 (34.8%)	6 (42.9%)	0.732
Concomitant therapies, n (%)			
Mesalamine	21 (91.3%)	13(92.9%)	1
Corticosteroids	7 (30.4%)	7 (50.0%)	0.303
Immunomodulators (azathioprine or 6-mercaptopurine)	11 (47.8%)	7 (50.0%)	1
Tacrolimus	3 (13.0%)	1 (7.1%)	1
Leukocytapheresis	8 (34.8%)	2 (14.3%)	0.26
partial Mayo score, median (interquartile range)	6 (5–7)	7 (5.25–7)	0.61
WBC (/μL), median (interquartile range)	5400 (4000–7700)	6550 (5880–7900)	0.199
Hemoglobin (g/dL), median (interquartile range)	11.3 (10.9–12.2)	12.0 (10.9–13.6)	0.301
Platelet (×10^4^/μL), median (interquartile range)	28.5 (23.6–31.8)	25.8 (30.0–31.5)	0.552
Albumin (g/dL), median (interquartile range)	3.70 (3.50–4.15)	3.85 (3.70–4.07)	0.469
CRP (mg/dL), median (interquartile range)	0.31 (0.07–1.29)	0.23 (0.04–0.42)	0.188
Neutrophil (/μL), median (interquartile range)	3730 (2400–4760)	4850 (4370–5790)	0.017
NLR, median (interquartile range)	2.46 (1.57–3.68)	5.78 (4.62–6.58)	0.003
Follow-up time, median (interquartile range) *months*	28.5 (14.7–45.8)	7.6 (5.2–12.9)	

UC: ulcerative colitis; WBC: white blood cell; CRP: C-reactive protein; NLR: neutrophil-to-lymphocyte ratio.

**Table 3:** Cox regression analysis of risk for loss of response during follow-up after induction of infliximab.

	No. of events	Rate	Unadjusted HR (95% CI)	*P*-value	Adjusted HR (95% CI)	*P*-value
Gender						
Male, n = 14	6	0.43				
Female, n = 23	8	0.35	0.66 (0.23–1.90)	0.437		
Age at diagnosis						
< 40 years, n = 24	9	0.38				
40 ≤ years, n = 13	5	0.38	1.06 (0.35–3.18)	0.911		
Age at start of infliximab						
< 45 years, n = 21	9	0.43				
45 ≤ years, n = 16	5	0.31	0.70 (0.23–2.09)	0.522		
Disease duration at initial dilation						
< 5.0 years, n = 15	6	0.40				
5.0 ≤ years, n = 22	8	0.36	0.83 (0.29–2.41)	0.739		
Disease location at initial dilation						
Left-sided colitis, n = 10	4	0.40				
Pancolitis, n = 27	10	0.37	1.11 (0.35–3.56)	0.859		
Prednisolone dependent						
No, n = 19	7	0.37				
Yes, n = 18	7	0.39	0.80 (0.28–2.31)	0.692		
Prednisolone resistant						
No, n = 23	8	0.35				
Yes, n = 14	6	0.43	1.54 (0.53–4.46)	0.427		
Mesalamine treatment at start of infliximab						
No, n = 3	1	0.33				
Yes, n = 34	13	0.38	0.40 (0.05–3.22)	0.386		
Steroid treatment at start of infliximab						
No, n = 23	7	0.30				
Yes, n = 14	7	0.50	1.81 (0.63–5.16)	0.268	1.61 (0.54–4.77)	0.389
Immunomodulators (azathioprine or 6-mercaptopurine) at start of infliximab						
No, n = 20	8	0.40				
Yes, n = 17	6	0.35	0.84 (0.29–2.42)	0.742		
Tacrolimus at start of infliximab						
No, n = 33	13	0.39				
Yes, n = 4	1	0.25	0.77 (0.10–5.88)	0.798		
Cytapheresis at start of infliximab						
No, n = 27	12	0.44				
Yes, n = 10	2	0.20	0.31 (0.07–1.40)	0.129	0.27 (0.06–1.29)	0.101
Severe ulcerative colitis (partial Mayo score ≥7) at start of infliximab						
No, n = 18	6	0.33				
Yes, n = 19	8	0.42	1.39 (0.48–4.00)	0.544		
Neutrophil-to-lymphocyte Ratio						
Low, n = 22	4	0.18				
High, n = 15	10	0.670	4.07 (1.28–13.0)	0.018	3.86 (1.20–12.4)	0.023

CI: confidence interval; HR: hazard ratio.

## Discussion

The principal finding of our study is that a high NLR is strongly and independently associated with an increased risk of LOR to infliximab in UC. Neutrophils, one of the most abundant and important mediators of innate immunity, are dedicated phagocytes which mount the acute inflammatory response and act as the first line of defense against invading pathogens [31]. Neutrophils are one of the most important leukocytes causing inflammation and tissue injury in UC disease [32]. Neutrophil accumulation and abscess formation within the intestinal crypts at the apical epithelial surface are typically seen in the pathological aspect of UC [33]. In fact, removal of activated circulating leukocytes or neutrophils by cytapheresis has been established as a therapeutic approach for UC [34]. Lymphocyte function in inflammatory bowel disease (IBD) is known to be abnormal at both the peripheral and the mucosal level [35], and the absolute lymphocyte count is assumed to reflect the degree of responsiveness of the host’s immune system [36]. Therefore, the NLR can be a biomarker that integrates two WBC subtypes, that is easily calculated from the differential WBC counts and less affected by conditions, representing two inversely related immune pathways. Unlike many other inflammatory markers, the NLR is universally available and inexpensive. Although neutrophil count may be affected by the use of corticosteroid, no significant difference in baseline NLR was found regardless of the corticosteroid use or corticosteroid dependency, and there was no correlation between baseline NLR and the dosage of corticosteroid. Furthermore, multivariate analysis including concomitant corticosteroid treatment revealed NLR was an independent prognostic factor for LOR.

A few previous studies illustrated the value of the NLR in patients with IBD. Two papers focused on the association between the NLR and the severity of the disease: They revealed that the NLR values of the active IBD group were elevated compared with those of the patients with inactive IBD [20, 21]. One study reported the significance of the NLR as a predictor of the sustained response to maintaining treatment in the 52-week therapy with infliximab in patients with Crohn’s disease [24]. However, no studies have been reported focusing on the relation between the NLR and the clinical course of infliximab treatment for patients with UC.

We have assessed the factors related to LOR in the patients who achieved clinical remission on infliximab. Comparison of clinical parameters between the two groups (sustained response versus LOR) indicates the NLR or absolute neutrophil count as possible risk factors for LOR. The NLR has the higher predictive value versus the absolute neutrophil count, based on an area under the ROC curve of 0.798. The best cut-off values for the NLR are greater than 4.488 (sensitivity: 78.6%, specificity: 78.3%). This result is similar to the results reported in a previous paper, which reveals that the NLR has a predictive value, and that the best cut-off value to predict a sustained response to infliximab treatment is 4.068, with 80% sensitivity and 87% specificity in Crohn’s disease treated with infliximab [24]. Considering the similar prognostic values of the NLR for infliximab treatment in patients with UC and Crohn’s disease, the NLR may be useful as a prognostic marker for other diseases treated with infliximab (e.g., rheumatoid arthritis or Behcet’s disease).

Several previous studies on the factors associated with LOR to infliximab in UC patients have reported that low trough levels of infliximab, presence of antibodies to infliximab, and high CRP values are risk factors for LOR [6, 37, 38]. The present study assesses the factors just before the induction of infliximab, in order to identify the useful predictors for selecting the proper treatment. We believe that the NLR may be more useful for predicting long-term outcomes on infliximab treatment, than trough levels or anti-infliximab antibodies, because the NLR can be assessed before the introduction of infliximab.

Both high- and low-NLR groups had similar primary responsiveness to infliximab in this study. No significant correlations were noted between baseline NLR and clinical activity, indicating that the concentration of infliximab, rather than the general activity of UC, might be associated with NLR in LOR to infliximab. One possible mechanism by which NLR might affect the clearance capability of infliximab, an immunoglobulin G1 monoclonal antibody, involves Fc-γ-receptor-mediated phagocytosis by cells such as neutrophils. [39, 40]. Patients with a higher baseline NLR may have a potentially higher clearance ability for infliximab, and be associated with a lower infliximab concentration. We are not able to estimate the influence of infliximab trough level in this study because of the retrospective study design. Further translational study is required to understand the mechanism.

Our study has some limitations. First, we could not rule out the possibility of the influence of infection on NLR. Although cardiovascular diseases, malignancies, or infection could affect NLR, no patients had symptoms related to cardiovascular diseases or malignancies in this study. Regarding infection, we do not usually measure procalcitonin levels, and it is difficult to determine the presence of bacteremia. However, both baseline NLR and NLR during maintenance periods, when patients should not have bacteremia, had value as predictors of LOR to infliximab; therefore, high NLR may be caused by disease/immune states but not by bacteremia. However, it was impossible to prove an association between bacteremia and baseline NLR in this study. The influence of bacteremia would be a limitation of the study. Second, we did not measure the serum trough infliximab levels and antibodies to infliximab in all patients prescribed infliximab; therefore, the relationship between NLR and serum trough level of infliximab could not be evaluated. Further study is needed to evaluate the association between infliximab trough level or infliximab autoantibody with NLR. Another limitation of this study is its retrospective nature, and a relatively small cohort. Therefore, a further large prospective study will help to confirm the NLR as a key predictor for infliximab treatment for UC.

In summary, the results of our study show that the baseline peripheral blood NLR that reflects a high neutrophil count, is an independent indicator of LOR to infliximab in patients with moderate-to-severe active UC. Taking NLR into account in patients with UC may lead to more appropriate clinical management of those patients, by indicating to the physicians that they should consider alternative treatments in patients with a high NLR.

## Supporting Information

S1 Fig The time course of neutrophil-to-lymphocyte ratio (NLR). A) Red line showed patients with loss of response and black line showed patients with sustained response; B) non-responders. No significant difference was found between baseline NLR and NLR at week 14 in those three groups (patients with loss of response: *P* = 0.173, patients with sustained response: *P* = 0.427, non-responders, *P* = 0.063, Wilcoxon rank sum test).
(TIF)

S2 Fig Comparison between baseline neutrophil-to-lymphocyte ratios (NLR) of patients with corticosteroid and patients without corticosteroid. Patients with corticosteroid treatment had tendency toward higher baseline NLR than patients without corticosteroid treatment (*P* = 0.057). The horizontal bar represents median value (A). There was no correlation between corticosteroid dosage and the baseline NLR (*r* = -0.438, *P* = 0.117) (B).
(TIF)

S3 Fig Association between baseline neutrophil-to-lymphocyte ratios (NLR) and partial Mayo score. There was no correlation between the NLR and the partial Mayo score(*r* = 0.0972, *P* = 0.567)
(TIF)

S1 Table The comparison of neutrophil-to-lymphocyte ratios (NLR) between patients with and without concomitant therapies. NLR: neutrophil-to-lymphocyte ratio.
(DOCX)
