# Supplementary material for: Neutrophil-to-Lymphocyte Ratio for Predicting Loss of Response to Infliximab in Ulcerative Colitis
Source: PLoS One. 2017 Jan 11;12(1):e0169845. doi: 10.1371/journal.pone.0169845 (PMC5226844; doi:10.1371/journal.pone.0169845)
Supplement: S1 Table — NLR: neutrophil-to-lymphocyte ratio. (DOCX) [file pone.0169845.s004.docx]

| Therapies (0; without, 1; with) | | n | NLR, median (interquartile range) | *P*-value |
| --- | --- | --- | --- | --- |
| Mesalamine | 0 | 3 | 1.41 (1.32–4.02) | 0.404 |
|  | 1 | 34 | 3.27 (2.00–5.50) |  |
| Corticosteroids | 0 | 23 | 2.56 (1.75–4.85) | 0.056 |
|  | 1 | 14 | 5.47 (2.51–6.57) |  |
| Immunomodulators | 0 | 20 | 2.63 (1.79–5.24) | 0.345 |
|  | 1 | 17 | 4.69 (2.40–5.74) |  |
| Tacrolimus | 0 | 33 | 3.36 (2.29–5.52) | 0.221 |
|  | 1 | 4 | 1.46 (1.20–4.90) |  |
| Leucocytapheresis | 0 | 27 | 3.19 (1.76–5.81) | 0.918 |
|  | 1 | 10 | 3.03 (2.47–5.16) |  |
